# Serological Investigations of Flavivirus Prevalence in Khammouane Province, Lao People's Democratic Republic, 2007–2008

**DOI:** 10.4269/ajtmh.2010.09-0480

**Published:** 2010-11-05

**Authors:** Alexandra Hiscox, Christian H. Winter, Phengta Vongphrachanh, Thongchanh Sisouk, Virasack Somoulay, Samlane Phompida, Surinder Kaul, Pany Sananikhom, Nguyen Thi Yen, Richard E. Paul, Paul Brey, Juliet E. Bryant

**Affiliations:** Institut Pasteur du Laos, Lao People's Democratic Republic; London School of Hygiene and Tropical Medicine, London, United Kingdom; Center for Malaria, Parasitology, and Entomology, Vientiane, Lao People's Democratic Republic; Nam Theun 2 Power Company, Vientiane, Lao People's Democratic Republic; National Institute of Hygiene and Epidemiology, Hanoi, Vietnam; Institut Pasteur, Paris, France; National Center for Laboratory and Epidemiology, Ministry of Health, Vientiane, Lao People's Democratic Republic

## Abstract

A large-scale cross-sectional seroprevalence study of dengue (DEN) and Japanese encephalitis (JE) was conducted in Khammouane province, Lao PDR, as part of the initial baseline health impact assessment of the Nam Theun 2 hydroelectric dam construction project. Health surveys were performed between May 2007 and February 2008 with serum samples collected from healthy individuals involved in the resettlement program of 16 villages (total surveyed population 4,369). Hemagglutination inhibition assay using flavivirus antigens (DENV1, DENV3, and JEV) performed on 1,708 plasma specimens revealed 30.4% (519) cross-reactive positives, and 10% (172) and 1.3% (22) positives to JEV or DENV, respectively. Entomological surveys conducted during the rainy season of 2008 indicated the presence of competent flavivirus vectors (*Culex vishnui* group and *Aedes albopictus*), although *Aedes aegypti* was not found. Continued surveillance and investigation is warranted to assess the clinical disease burden of flaviviruses in this area that is undergoing rapid ecological and demographic change.

Construction of large dams for the generation of hydroelectricity and irrigation is an active area of economic development in the Lao People's Democratic Republic (PDR). The Nam Theun 2 Hydroelectric Project is located in the south-central part of Laos on the Nakai Plateau (elevation: ~500 m) situated between Thailand and Viet Nam (latitude 105°10′, longitude 17°40′) (Figure 1). Construction began in 2005 and inundation of the reservoir took place throughout the rainy season of 2008 with the hydroelectric plant becoming operational in early 2010. During the construction phase extensive logging to clear future reservoir area of 450 km^2^ took place and 150 km of new roads were constructed, providing opportunity for large demographic shifts on the Nakai plateau. Creation of the reservoir required relocation of ~7,000 people (1,462 households) into 16 resettlement villages.

**Figure 1.**
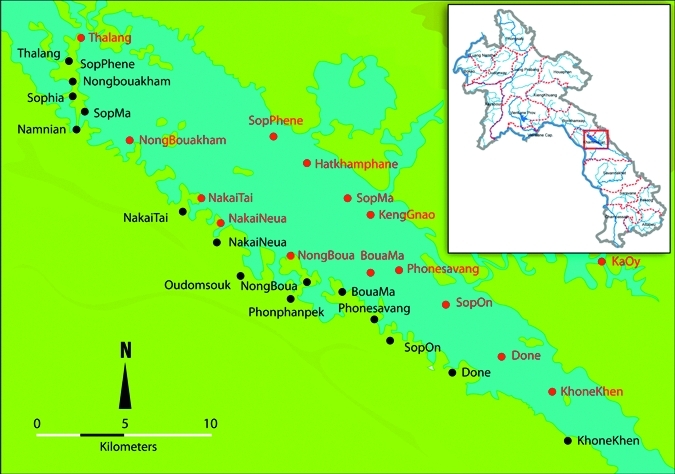
Map of Nam Theun reservoir showing locations of original villages before inundation (red) and resettlement areas (black).

Alongside concerns about the social and environmental impacts of the dam, there is interest in monitoring potential changes in the prevalence of vector-borne diseases, which may be affected by modifications to mosquito breeding sites and altered patterns of interaction between people and vectors. Although a substantial body of literature exists regarding the impact of large dams on malaria transmission,^1^ relatively little is known about how hydroelectric projects may alter arbovirus epidemiology. Of key interest is the potential for changes in transmission of dengue virus (DENV) and Japanese encephalitis virus (JEV), both of which are flaviviruses endemic to Laos and are considered national public health priorities. Here, we present the results of a baseline cross-sectional epidemiological serosurvey conducted within the resettlement population at the time of relocation, and baseline entomological surveys carried out on the Nakai plateau. The collections of these baseline data are critical for monitoring future changes in population exposure rates, shifting demographics of infection, and vector bionomics. Additional cross-sectional sampling and entomological sampling is planned for 2010.

In the Lao PDR, dengue cases are reported from most provinces of the country every year, although surveillance is limited to hospitalized cases of which only a small fraction are laboratory-confirmed. Previous epidemiological studies of dengue transmission in Lao PDR have indicated high levels of endemicity in both urban^2^–^4^ and rural communities,^5^ with reported dengue immunoglobulin G (IgG) seroprevalence in adults from 79% to 88%. The first confirmed case of Japanese encephalitis (JE) reported from Laos was in 1989,^6^ but to date no systematic surveillance has ever been conducted. Although vaccines against JEV are available, vaccination is not currently part of the national immunization program. Erlanger and others^7^ classify Laos as a country in which the incidence of JEV seems to have increased recently and where disease surveillance is inadequate. The principle vector of DENV, *Aedes aegypti*, is present throughout the country, as are competent vectors for JEV, the *Culex vishnui* group. To date, DENV and JEV are the only known flavivirus species that have been identified from Laos; however, there have never been any investigations to identify or isolate less known flavivirus species.

In this study, we evaluated serum samples from healthy individuals in 10 rural resettlement villages on the Nakai plateau. The serum samples had been collected from individuals participating in Health Checks and Surveys conducted by the Provincial Health Services and the Nam Theun 2 Power Company (NTPC) Health Program Management Unit at the time of village resettlement. The project objectives were explained to villagers and informed consent was obtained orally in Lao language during community education campaigns held in conjunction with the health checks, and written consent was obtained from Village Heads. Each individual who agreed to participate in the study was administered a questionnaire that included assessment of demographics, education, occupation, medical history, and social behavior relevant to health. The study protocol was approved by the Lao National Ethics committee and by the Institut Pasteur Clinical Research Committee. Additional approval for entomological work was granted by the Lao National Center for Malaria, Entomology and Parasitology, and the London School of Hygiene and Tropical Medicine ethics committee.

A total of 3,040 sera specimens were collected and 1,744 were processed by hemagglutination inhibition (HI) assay. Sample processing was comprehensive for all surveyed villages with the exception of the two largest villages, for which 15% (94/620 samples) and 29.9% (96/321) of available specimens were processed. Of the 1,744 tests performed, 36 test results were omitted because of errors in specimen identification or incomplete demographic data. The HI assay was carried out in the conventional manner^8^ using sucrose-acetone extracted antigens for DENV1, DENV3, and JEV provided by Institut Pasteur of Cambodia. The HI was the method of choice for this study because it was not feasible to conduct neutralization tests in-country, and because the HI assay enabled parallel serial titrations against both DENV and JEV antigens to assess the relative reactivity to these representative flaviviruses. Samples were considered positive if they produced an HI reactive titer > 1:40. Serum samples positive for both DENV and JEV were scored as “flavivirus positive.” Samples were scored as either “DENV positive” or “JEV positive” if they produced a 2-fold higher titer to the homologous virus. Odds ratio (OR) analyses were performed with Stata 9 (Stata Corp., LP, College Station, TX).

The overall prevalence of flavivirus reactive antibody was 41.7%. A total of 30.4% (519) serum samples were positive for both DENV and JEV, whereas 10% (172) were positive to just JEV and 1.3% (22) were positive to just DENV. Overall seropositivity increased significantly with age (Figure 2), as confirmed by increased ORs for risk of infection for each age group > 10 years of age. The seropositivity of children < 5 years of age was 20%, 5 to < 10 years 21.4%, 10 to < 15 years 29.6%, and for people older than 15 years the seropositivity was 51.2%. Persons 30 years of age or older had more than a 2-fold higher risk of being seropositive (OR 2.12, 95% confidence interval [CI] 1.64–2.75) as compared with persons younger than 30 years of age. Increased seropositivity with age is expected for endemic areas, as shown by numerous previous studies,^9^–^11^ and is consistent with the fact that IgG antibodies against flaviviruses persist for many years. These results confirm that one or more flavivirus species are endemic to the resettlement area. Given the extensive cross reactivity of flaviviruses and the limitations of the HI assay, these data are not sufficient to reliably distinguish which flavivirus species was responsible for the observed background immunity of the population. Indeed, the possibility that non-DENV or non-JEV flaviviruses are circulating in the area cannot be ruled out. Furthermore, as with all pathogens that show highly variable clinical-to-subclinical ratios, translation of the observed flavivirus seroprevalence into actual measures of morbidity is difficult. No cases of either suspected dengue or encephalitis were reported from the communities during 2007 or 2008, suggesting that the majority of previous flavivirus infections were mild or asymptomatic. However, it is possible that positive cases were missed, because clinical expertise and diagnostic facilities are still very limited within the communities. Interestingly, seroprevalence in this study was lower than observed in previous Lao serosurveys in Attapeu province (79%)^5^ and urban Vientiane (57.7%).^4^ Our seroprevalence rates also appear lower than reports from neighboring Southeast Asian countries, such as Southern Vietnam (Binh Thuan Province) showing 53% dengue IgG prevalence among children 7 years of age,^12^ and a study from Thailand (Mueang district, Ratchaburi Province) showing 69% dengue IgG prevalence among children (5–12 years of age).^13^

**Figure 2.**
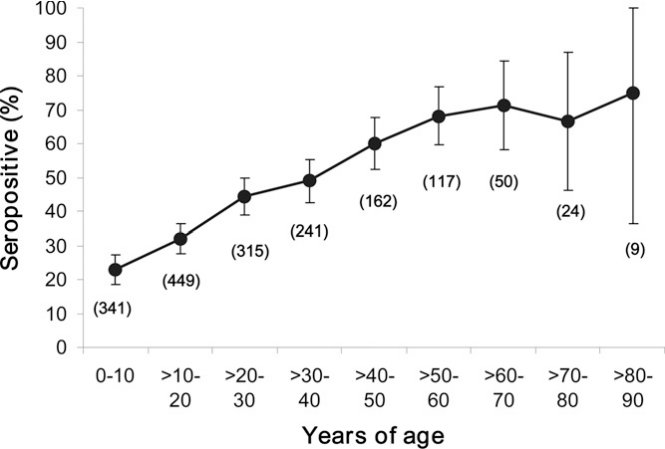
Seroprevalence of anti-flavivirus antibodies of different age groups, including 95% confidence interval (*N* = sample size per age group).

The geographic distribution of flavivirus seropositives within the resettlement area varied between villages, from 18.4% to 79.7% (Figure 3). The baseline data presented here reflects exposures to flaviviruses that most likely occurred at the original village locations; unfortunately, descriptive data regarding the original village sites was not available. The villages with the lowest seroprevalence were located at the center of the reservoir, whereas the villages with higher seroprevalence were located at either end of the reservoir. We speculate that variation in factors such as land cover, numbers of domestic livestock, and housing density, or varying patterns of human travel may influence arbovirus eco-epidemiology; these factors along with other socio-economic variables will be studied in future cross-sectional surveys.

**Figure 3.**
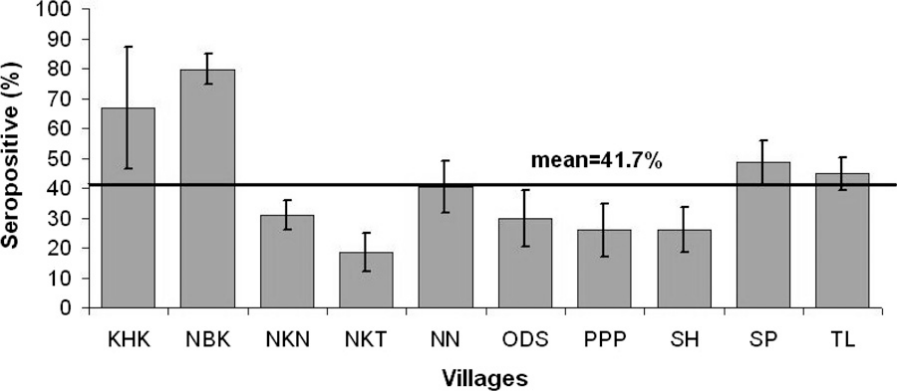
Seroprevalence (mean and 95% confidence interval) of anti-flavivirus antibodies of 10 different villages. KHK, Old SopHia Phonetong; NBK, NongBouakham; NKN, NakaiNeua; NKT, NakaiTai; NN, Namnian; ODS, Oudomsouk; PPP, Phonphanpaek; SH, SopHia, SP, SopPhene; TL, Thalang.

Mosquito trapping was performed throughout the rainy season of 2008 (July to September) in peri-domestic settings of two representative villages using Centers for Disease Control and Prevention (CDC) light traps (*N* = 12). Traps were set overnight beside occupied bed nets inside houses, in animal sheds, and in latrines close to resettlement houses, for a total of 292 trap nights. Additional mosquito sampling using a cattle-baited trap was conducted in one village over 14 nights in September. A short, intensive, survey was conducted in a further four villages over 1 week at the end of September 2008 using CDC light traps, cattle-baited traps, and hand-held aspirators. Larval container surveys were conducted at this time in 20 to 25 randomly selected households per village as previously described,^14^ and data were entered into the Vector Productivity Data Calculator (version 5.0, AFAP) for calculation of the World Health Organization (WHO) vector density indices. Average daytime and nighttime temperatures and relative humidity throughout the study period remained fairly constant (27.8°C, 22.6°C, and 85%, respectively) and total rainfall between July and September was 1,425 mm (data provided by Nam Theun 2 Power Company). Adults and larvae were identified morphologically using illustrated keys to the mosquitoes of Thailand^15^–^17^ and keys to the subgenus *Stegomyia* of *Aedes* in Southeast Asia.^18^ Confirmation of identification was carried out by entomologists from National Institute for Hygiene and Epidemiology/Hanoi.

Inclusive of all trapping methods and study villages, a total of 9,298 adult mosquitoes were collected during the rainy season of 2008 (sampling between July and September). Table 1 shows the proportion that each species contributed to the total catch. The most abundant species in the resettlement area were *Anopheles barbirostris* group (mostly *An. barbirostris*), *Culex vishnui* group (mostly *Cx. tritaeniorhynchus*), and *Armigeres subalbatus. Aedes albopictus* immature stages were sampled during container surveys in December 2007 and September 2008, but larvae of *Ae. aegypti* have not yet been found in the resettlement area. The types of containers most commonly infested with *Ae. albopictus* larvae were discards (general debris, used tires, and plastic waste), uncovered metal drums and large rain water collectors used before the installation of deep fresh water wells in the resettlement villages. Larval density indices varied between the villages surveyed (House index from 25% to 45%; Breteau index from 25 to 65; Container index from 7% to 20%). The apparent absence of *Ae. aegypti* in the resettlement area requires further study because this species is abundant in the provincial capital of Thakhek,^19^ located 90 km from the resettlement area.

The serological and entomological findings presented here confirm the importance of continued surveillance activities to monitor the changing ecology of vector-borne diseases in the area. Further studies will be required to more clearly identify which flavivirus species are responsible for the background immunity of the population, and to assess the potential public health significance of flavivirus circulation. Development efforts are currently focusing on improving health services that will enable improved diagnosis, treatment, and reporting of fever cases. Additional cross-sectional sampling of the population is planned for 2010, as well as continued vector studies, and virological analysis of specimens from wild and domestic animal populations. Entomological studies are already underway to assess the relationship between vector bionomics and improved housing and sanitation conditions in the resettlement area.

## Figures and Tables

**Table 1:** Mosquito species composition in resettlement villages (female mosquitoes collected between July and September 2008)

Proportion of total catch (%)*			
Species	CDC light trap	Cattle-baited trap	Light trap and CBT combined
	(*N* = 3,878)	(*N* = 2,586)	(*N* = 6,464)
*Aedes albopictus*	0.6	0	0.4
*Anopheles barbirostris* group	23.4	45.3	32.1
*An. hyrcanus* group	2.6	2.1	2.4
*An. philippinensis*	0.4	1.1	0.7
*An. sinensis*	2.2	1.5	1.9
Other *Anopheles*, spp.	0.8	2.1	1.3
*Armigeres subalbatus*	8.6	7.3	8.1
*Culex bitaeniorhynchus*	4.5	2.9	3.8
*Cx. fuscocephala*	1.7	0.5	1.3
*Cx. gelidus*	0.3	0	0.2
*Cx. hutchinsoni*	0.7	2.5	1.4
*Cx. pseudovishnui*	0.5	0.3	0.4
*Cx. quinquefasciatus*	2.4	0.2	1.5
*Cx. tritaeniorhynchus*	25.5	24.8	25.2
*Cx. vishnui*	7.9	1.2	5.2
*Cx. whitmorei*	14.7	5.5	11
Other *Culex*, spp.	3.1	2.5	2.9
Other mosquito genera	0.2	0.2	0.2

* CDC = Centers for Disease Control and Prevention; CBT = cattle-baited trap.
